# Lymphangiogenesis and Axillary Lymph Node Metastases Correlated with VEGF-C Expression in Two Immunocompetent Mouse Mammary Carcinoma Models

**DOI:** 10.4061/2011/867152

**Published:** 2011-10-19

**Authors:** Yuko Ito, Masa-Aki Shibata, Nabil Eid, Junji Morimoto, Yoshinori Otsuki

**Affiliations:** ^1^Division of Life Sciences, Department of Anatomy and Cell Biology, Osaka Medical College, 2-7 Daigaku-Machi, Takatsuki, Osaka 569-8686, Japan; ^2^Laboratory of Anatomy and Histopathology, Faculty of Health Science, Osaka Health Science University, 1-9-27 Temma, Kita-ku, Osaka 530-0043, Japan; ^3^Laboratory Animal Center, Osaka Medical College, 2-7 Daigaku-Machi, Takatsuki, Osaka 569-8686, Japan

## Abstract

Lymphangiogenesis and the expression of vascular endothelial cell growth factor C (VEGF-C) in tumors have been considered to be causally promoting lymphatic metastasis. There are only a few studies on lymphatic metastasis in immunocompetent allograft mouse models. To study the relationship between VEGF-C-mediated lymphangiogenesis and axillary lymph node metastasis, we used two mouse mammary carcinoma cell lines; the BJMC338 has a low metastatic propensity, whereas the BJMC3879 has a high metastatic propensity although it originated from the former cell line. Each cell line was injected separately into two groups of female BALB/*c* mice creating *in vivo* mammary cancer models. The expression level of VEGF-C in BJMC3879 was higher than BJMC338. As the parent cell line, BJMC3879-derived tumors showed higher expression of VEGF-C compared to BJMC338-derived tumors. This higher expression of VEGF-C in BJMC3879-derived tumors was associated with marked increase in infiltrating macrophages and enhanced expression of lymphatic vessel endothelial hyaluronan receptor-1 (LYVE-1) reflecting increased tumoral lymphatic density and subsequent induction of axillary lymph node metastasis. Our mouse mammary carcinoma models are allotransplanted tumors showing the same axillary lymph node metastatic spectrum as human breast cancers. Therefore, our mouse models are ideal for exploring the various molecular mechanisms of cancer metastasis.

## 1. Introduction

Based on clinical and pathological observations in human mammary carcinomas, the metastatic spread of mammary carcinoma cells is responsible for the majority of cancer deaths [1–5]. The common pathway of initial cancer dissemination is via lymphatics due to their characteristic endothelial structure with blind-ending capillaries [6]. In addition, metastasis to the regional lymph nodes through the lymphatic vessels is considered to be a common step in the progression of cancer and an important prognostic factor in many types of cancer including breast carcinomas. Lymphatic vessel density (LVD) in many types of solid cancer is associated with lymph node metastasis or poor prognosis, as has been reported in experimental and clinical studies [1–5, 7, 8]. Although mammary carcinoma is well known to have the character for lymph node metastasis, there are only a few mouse mammary carcinoma models showing extensive metastasis to lymph nodes. The chick embryo chorioallantoic membrane [9] and immunodeficient mice, SCID mice, or nude mice were used as xenotransplanted host animals to examine metastasis [10–12]. Recent studies have shown the important roles of tumor-associated macrophages (TAMs) expressing CD68 in cancer-mediated lymphangiogenesis [13–15]. Accordingly, the mouse immunocompetent model appears to be necessary for studying lymphangiogenesis and lymph node metastasis.

 The vascular endothelial growth factor C (VEGF-C) is a major lymphangiogenic factor. There is some evidence that VEGF-C promotes lymphangiogenesis under several normal and pathological conditions [16]. In VEGF-C-deficient mouse embryos, lymphatic vessels fail to develop from veins [17] resulting in prenatal death owing to fluid accumulation in the tissues of mouse embryos and edema in adults [18]. On the other hand, in VEGF-C transgenic mice, hyperplasia of lymphatic vasculature has been reported [19, 20].

To study the relationship between lymphangiogenesis mediated by VEGF-C and axillary lymph node metastasis, two-mouse mammary carcinoma cell lines with different metastatic properties were used in this study. Both cell lines were derived from the same BALB/*c* mouse, one of them, the BJMC338 cell line, an adenocarcinoma cell line, has a low metastatic propensity, whereas the other cell line, BJMC3879, has a high metastatic propensity, particularly to lymph nodes and lungs [21]. Each cell line was injected separately into two groups of adult female BALB/*c* mice creating *in vivo* mammary cancer models. In this inoculation study, we found that increased expression of tumor-derived VEGF-C correlates with LVD and axillary lymph node metastasis. 

## 2. Materials and Methods

### 2.1. *In Vitro* Studies

#### 2.1.1. Cell Culture and Cell Preparation

 Two-mouse mammary carcinoma cell lines were used in this study. The BJMC338 mammary adenocarcinoma cell line used in this study was derived from a female BALB/*c* mouse infected with mouse mammary tumor virus (MMTV) into the inguinal mammary glands and shows low metastatic property. The BJMC3879 mammary adenocarcinoma cell line was derived from foci within metastatic lymph node and lung of a female BALB/*c* mouse that had been injected with BJMC338 cell line into the right inguinal region. The BJMC3879 mammary cell line shows a high metastatic propensity, especially to lymph nodes and lungs [22]. Both cell lines were maintained in RPMI 1640 medium containing 10% fetal bovine serum (FBS) with streptomycin/penicillin in an incubator under 5% CO_2_. The cells were immediately rinsed with 0.01 M phosphate buffered saline (PBS), fixed with 2% paraformaldehyde and 2.5% glutaraldehyde in 0.1 M phosphate buffer (PB) (pH 7.4) for 1 h, for transmission electron microscopy (TEM). Cells were subsequently postfixed in 1% OsO_4_ for 45 min at room temperature, dehydrated in a series of graded ethanol concentrations, cleared in propylene oxide, and embedded in an epoxy resin mixture. For immunofluorescent study, cells were fixed with 1% paraformaldehyde in PBS for 10 min.

#### 2.1.2. TEM

 After preparation, ultrathin sections were prepared and stained with uranyl acetate and lead citrate. Sixty nm sections were examined by TEM using H-7100 and H-7650 (Hitachi, Tokyo, Japan).

#### 2.1.3. Immunofluorescent Study

 Following fixation, cells were transferred to PBS for 15 min, followed by exposure to 1% Block Ace (Dainippon Sumitomo Pharma Co., Ltd., Tokyo, Japan) for 20 min to block nonspecific antibody binding. Cells were then incubated with a primary rabbit anti-VEGF-C antibody (rabbit polyclonal; Santa Cruz Biotechnology, Santa Cruz, Calif, USA) for 1 h at room temperature (RT). After rinse in PBS for 15 min, cells were incubated with a secondary fluorescein-isothiocyanate- (FITC-) conjugated anti-rabbit antibody (Dako, Carpenteria, Calif, USA) for 30 min at RT. Samples were counterstained with propidium iodide (PI) for 15 min and observed under a model Radiance 2000 MP confocal scanning laser microscopy (Bio-Rad, Hercules, Calif, USA).

#### 2.1.4. Western Blot Analysis for VEGF-C Protein

 Samples containing 20 *μ*g of protein from cultured cells and tumors were fractionated in 10% Tris-glycine gels under reducing conditions and transferred onto nitrocellulose membrane. Anti-VEGF-C antibody (Santa Cruz Biotechnology) was applied to membranes, incubated with appropriate horseradish peroxidase-conjugated secondary antibody, and visualized on X-ray films using enhanced chemiluminescence (Perkin Elmer Life Science, Inc., Boston, Mass, USA).

#### 2.1.5. RNA Preparation and Reverse Transcription-Polymerase Chain Reaction (RT-PCR)

 Total RNA was isolated from cultured BJMC338 and BJMC3879 cells using an RNeasy Mini Kit (Qiagen, Hilden, Germany) and cDNA Synthesis Kit (Roche Diagnostic Kit, Hilden, Germany) following the manufacturer's protocol, with the total mRNA concentration adjusted to 5 *μ*g/*μ*L in each sample. Primer sequences for VEGF-C were 5′-CCTTCTTTAAACCTCCATGTGT-3′ and 5′-GCAAAACTGATTGTGACTGGT-3′; for glyceraldehyde-3-phosphate dehydrogenase (GAPDH) used as internal control 5′-TGCACGGGAAGCTCACTGG-3′, 5′-TCCACCACCCTGTTGCTGTA-3′ (Nihon Gene Research Laboratories, Sendai, Japan). Products were amplified in a GeneAmp PCR System 9700 (Applied Biosystems, Foster City, Calif, USA) with preincubation at 98°C for 3 min followed by 30 cycles of 94°C for 30 s, 58°C for 30 s, and 72°C for 60 s. The amplification was finished with a single-3 min incubation at 72°C. The PCR products were separated on 1.5% agarose gels, stained with 0.1 mg/mL ethidium bromide, visualized by UV transillumination, and documented on black and white instant films.

### 2.2. *In Vivo* Studies

#### 2.2.1. Animals and Preparation of Tumors

 A total of 40 female 6-week-old BALB/*c* mice (Japan SLC Inc., Hamamatsu, Japan) were used in this study. All manipulations of mice were performed in accordance with the procedures outlined in the Guide for Care and Use of Laboratory Animals in Osaka Medical College. Mice were divided into two groups (20 mice each), and BJMC338 and BJMC3879 cells (5 × 10^6^ cells/0.3 mL in PBS) were inoculated subcutaneously into right inguinal region, respectively. Mice were sacrificed at 4, 6, 8, 10 weeks after inoculation under ether anesthesia. Tumor samples were frozen for Western blot assay and RT-PCR. Another one was fixed for histopathology and immunohistochemistry with 4 or 10% paraformaldehyde, and for electron microscopy in a same fixative as *in vitro* study. Lungs and right axillary lymph nodes of mice were removed and then fixed as same as tumors. Samples fixed with paraformaldehyde were processed through to paraffin embedding.

#### 2.2.2. Histopathology and Immunohistochemistry

To examine histopathology and metastasis, tumors, lungs, and light axillary lymph nodes were stained with Hematoxylin and Eosin (H&E). Ultrastructural features of tumors were checked under TEM. The avidin-biotin complex method was used for immunohistochemistry. To visualize lymphatic vessels, tumors were stained with primary antibodies to the lymphatic-specific marker, lymphatic vessel endothelial hyaluronan receptor-1 (LYVE-1) (rabbit polyclonal, Acris Antibodies GmbH, Hiddenhausen, Germany). VEGF-C (Santa Cruz) was labeled on tumor sections, and activated macrophages were demonstrated by CD68 antibody (rat anti-mouse CD68, AbD Serotec, Oxford, UK).

#### 2.2.3. Lymphatic Vessel Density (LVD) and Counting of Macrophage

The number of lymphatic vessels immunolabeled with anti-LYVE-1 antibody in tumor sections at 4 to 10 weeks postinoculation as well as the number of CD68-positive cells in tumor sections at 4 weeks postinoculation were counted under light microscopy at higher magnification (×200).

#### 2.2.4. Statistical Analysis

The above-mentioned data were analyzed by Student's *t*-test. *P* < 0.05 was considered statistically significant.

## 3. Results

### 3.1. *In Vitro* Studies

#### 3.1.1. Ultrastructure and Expression of VEGF-C

**Figure 1:**
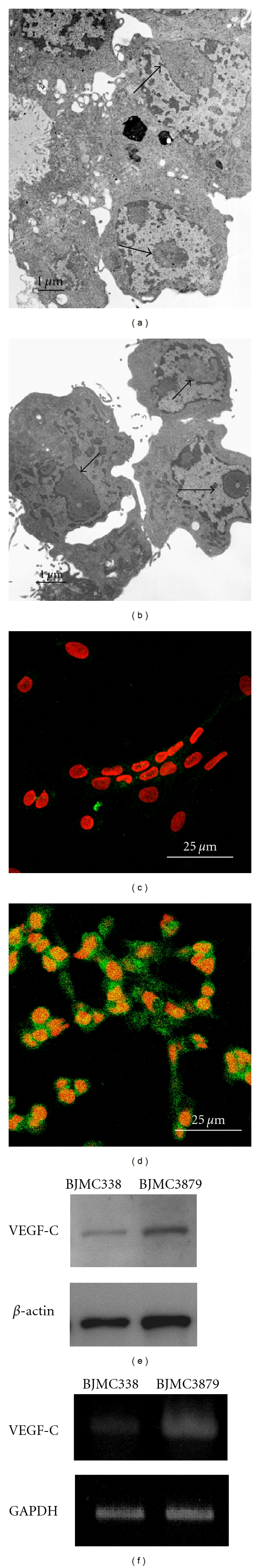
TEM micrographs (a and b), immunofluorecent staining (c and d), Western blot analysis (e), and RT-PCR analysis (f) of VEGF-C in the two-mouse mammary carcinoma cell lines. Both BJMC338 cells (a) and BJMC3879 cells (b) have the similar ultrastructure. BJMC338 cells (c) have minimally any activity of VEGF-C, whereas BJMC3879 cells (d) have moderate expression. Western blot analysis (e) demonstrates the same activity of VEGF-C as in (c and d). VEGF-C mRNA expression in BJMC3879 cells is higher than that of BJMC338 cells (f). Green fluorescence (FITC) indicates activity of VEGF-C, and red fluorescence (PI) shows nuclei of cells in (c and d).

 Ultrastructural differences between BJMC338 and BJMC3879 cells were investigated using TEM. Both cell lines showed the same morphology under TEM (Figures 1(a) and 1(b)). They had prominent nucleoli and dispersed small condensed chromatin in their nuclei. By immunofluorescent study, VEGF-C was barely expressed in BJMC338 cells, whereas moderately expressed in BJMC3879 cells (Figures 1(c) and 1(d)). The levels of VEGF-C protein in the two cell lines were determined by Western blot; the intensity of the bands was measured and corrected against **β**-actin intensity. A moderate increase in the VEGF-C protein level was detected in BJMC3879 cells (Figure 1(e)). RT-PCR analysis showed higher VEGF-C mRNA expression in BJMC3879 cells than in BJMC338 cells (Figure 1(f)).

### 3.2. *In Vivo* Studies

#### 3.2.1. Histopathology and Metastasis

**Figure 2:**
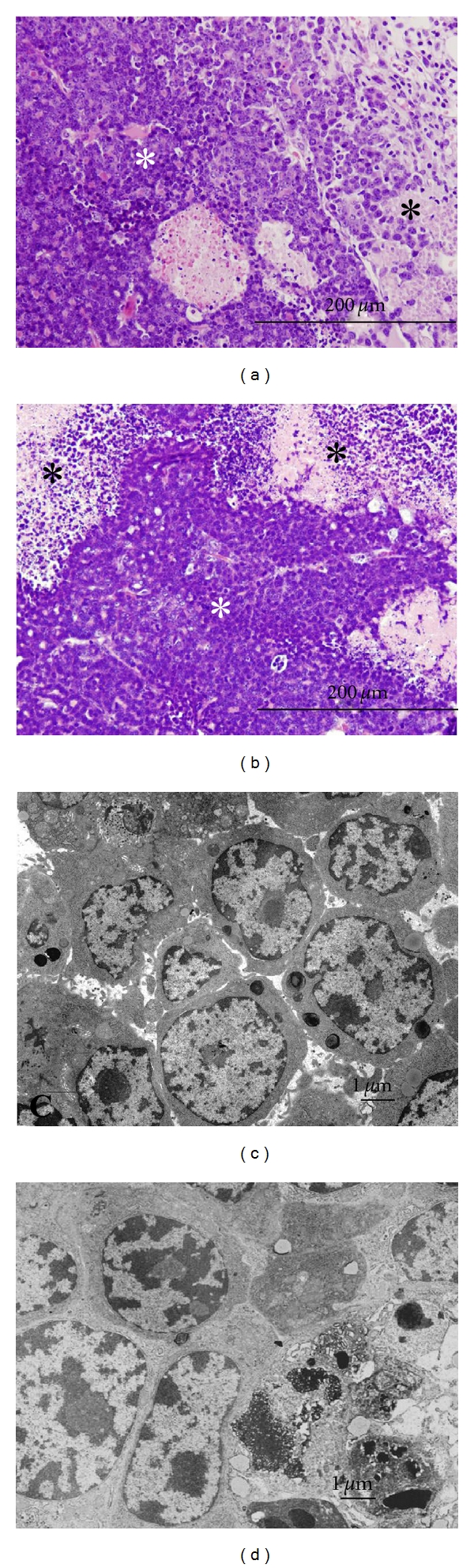
Histopathology of the inoculated tumors at 10 weeks postinoculation. H&E staining (a and b) show viable region (white ∗) and necrotic region (black ∗) in BJMC338 tumor (a) and BJMC3879 tumor (b). TEM micrographs (c and d) indicate tumor cells in the viable region of BJMC338 tumor (c) and BJMC3879 tumor (d).

**Figure 3:**
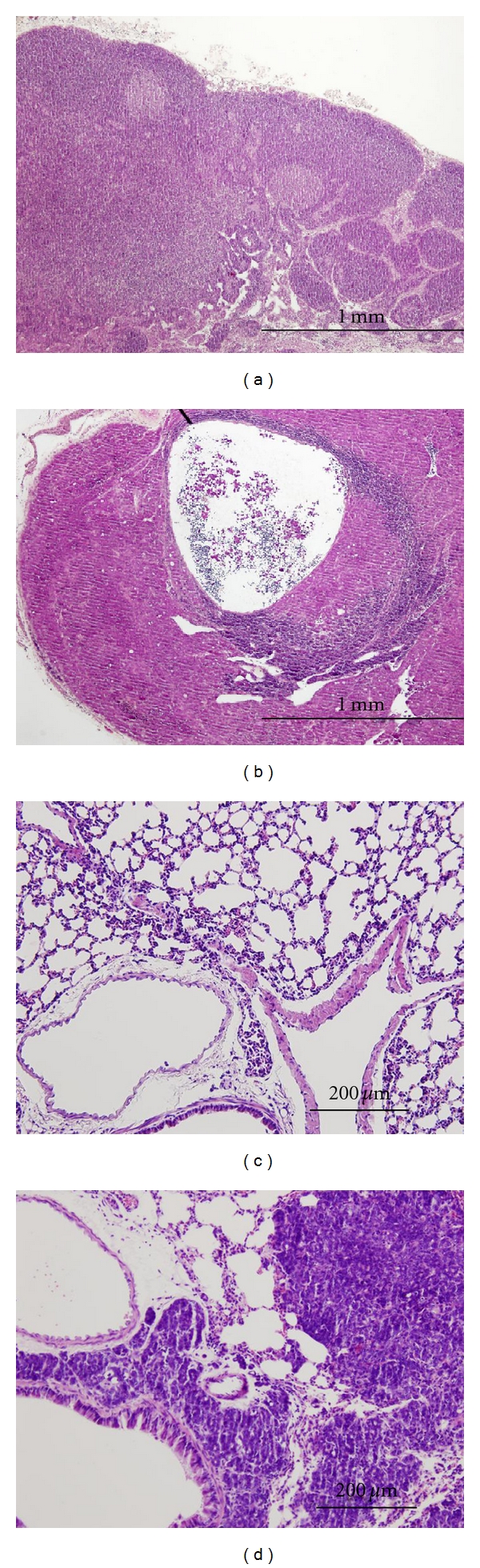
Histopathology by H&E staining of axially lymph nodes (a and b) and lungs (c and d) in mice at 10 weeks postinoculation. Axially lymph node and lung in BJMC338 tumor (a and c) show normal appearance, whereas those of BJMC3879 tumor (b and d) demonstrate metastatic foci.

Histopathologically, the two types of inoculated mammary carcinoma (BJMC338 and BJMC3879 tumors) proved to be moderately differentiated adenocarcinomas. Both tumors were accompanied by a viable region, a central necrosis, and an inflammatory region (Figures 2(a) and 2(b)). The morphology of the tumor cells in the viable region was the same as that of the cultured cell lines (Figures 2(c) and 2(d)). Metastasis to axillary lymph nodes or lungs at 8 and 10 weeks postinoculation was validated by the observation of sections stained with H&E. At 8 and 10 weeks postinoculation, no metastasis was observed in the lymph nodes and lungs of mice that were inoculated with BJMC338 cells (Figures 3(a) and 3(c)). In contrast, all the mice inoculated with BJMC3879 cells showed distant metastasis to axillary lymph nodes and lungs at 8 and 10 weeks postinoculation (Table 1, Figures 3(b) and 3(d)). 

#### 3.2.2. Lymphangiogenesis in Tumor Mouse Models

**Figure 4:**
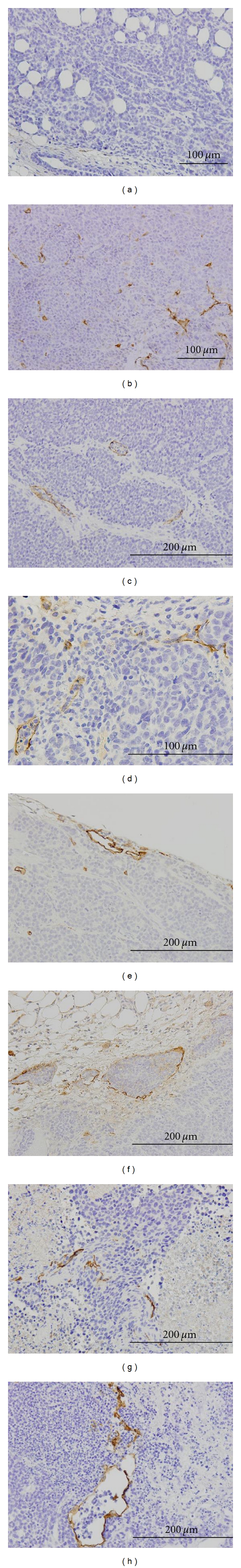
Immunohistochemistry of LYVE-1 in BJMC338 tumors (a, c, e, and g) and BJMC3879 tumors (b, d, f, and h) at 4 weeks (a and b), 6 weeks (c and d), 8 weeks (e and f), and 10 weeks postinoculation (g and h). Note the upregulation of LYVE-1 in BJMC3879 tumors.

 The lymphatic vessels in the tumor were detected by immunohistochemistry using the antibody specific to lymphatic vessels, LYVE-1. At 4 weeks postinoculation, few lymphatic vessels were found in BJMC338 tumors (Figure 4(a)). At 6 weeks postinoculation, several lymphatic vessels were observed in intratumoral connective tissues and/or surrounding connective tissues (Figures 4(c), 4(e), and 4(g)). Conversely, large numbers of dilated lymphatic vessels with or without tumor cells were observed within and around BJMC3879 tumors at 4 to 10 weeks postinoculation (Figures 4(b), 4(d), 4(f), and 4(h)).

#### 3.2.3. Lymphatic Vessel Density (LVD)

**Figure 5:**
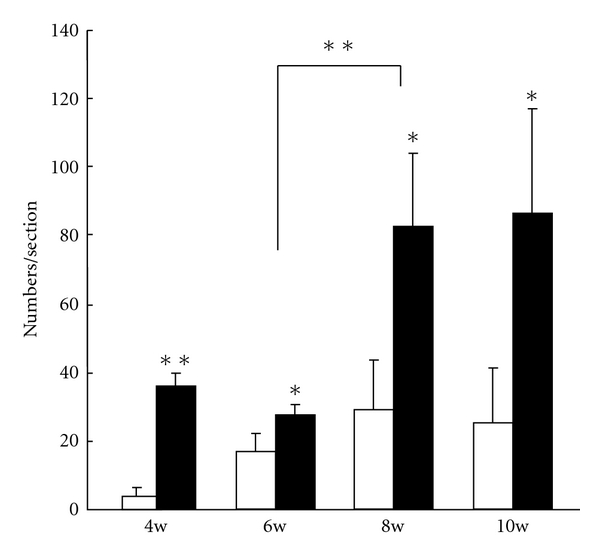
Density of lymphatic vessels (LVD) in tumors. The LVD in BJMC3879 tumors (black bars) is always significantly higher than that of BJMC338 tumors (white bars) (*P < 0.01, **P < 0.001).

The LVD of BJMC3879 tumors was always significantly higher than that of BJMC338 tumors (Figure 5). In BJMC338 tumors, the LVD at 6 weeks postinoculation was significantly higher than that at 4 weeks postinoculation (*P* < 0.01), after that, no significant difference in the LVD was observed (Figure 5). However, in BJMC3879 tumors, the difference in the LVD between 8 and 10 weeks postinoculation was not significant; a significant difference was detected between 6 and 8 weeks postinoculation (*P* < 0.001), namely, the LVD markedly increased at 8 weeks postinoculation (Figure 5).

#### 3.2.4. TEM for Lymphatic Vessels

**Figure 6:**
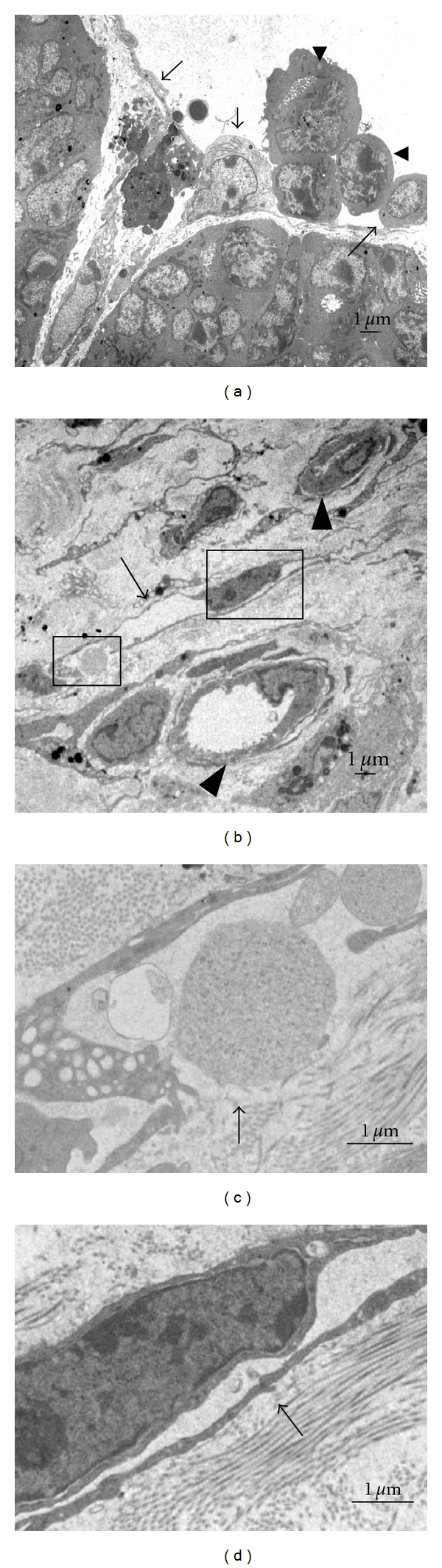
TEM micrographs of lymphatic vessels in BJMC3879 tumors at 10 weeks postinoculation (a) and 4 weeks postinoculation (b). The boxed areas in (b) are observed with high-power view (c and d). (a) A leukocyte (∗) is seen between endothelial cells (arrows), whereas tumor cells (arrowheads) are observed in the lumen. (b) Lymphatic capillary (arrow) and blood capillaries (arrowheads) are detected in the connective tissue surrounding tumors. Casein-like droplet (arrow) is located in the opening junction (c), and characteristic over-lapping junction (arrow) is showed (d).

 The status and ultrastructural features of lymphatic vessels in inoculated tumors were examined under TEM. Lymphatic capillaries were observed in the connective tissue surrounding and inside the tumors. They were distinguished from blood capillaries. The examination of lymphatic capillaries revealed that they had thin endothelium, abluminal protrusion of endothelial cells, no pericyte, no lamina densa, and overlapping junctions (Figures 6(a)–6(d)). Intraluminal tumor cells were observed in their lumen (Figure 6(a)). Interestingly, a leukocyte was shown to go into and/or out from the lymphatic lumen (Figure 6(a)). Furthermore, the absorption of caseinlike droplets into the lymphatic lumen was observed (Figure 6(c)). 

#### 3.2.5. VEGF-C Expression in the Tumors

**Figure 7:**
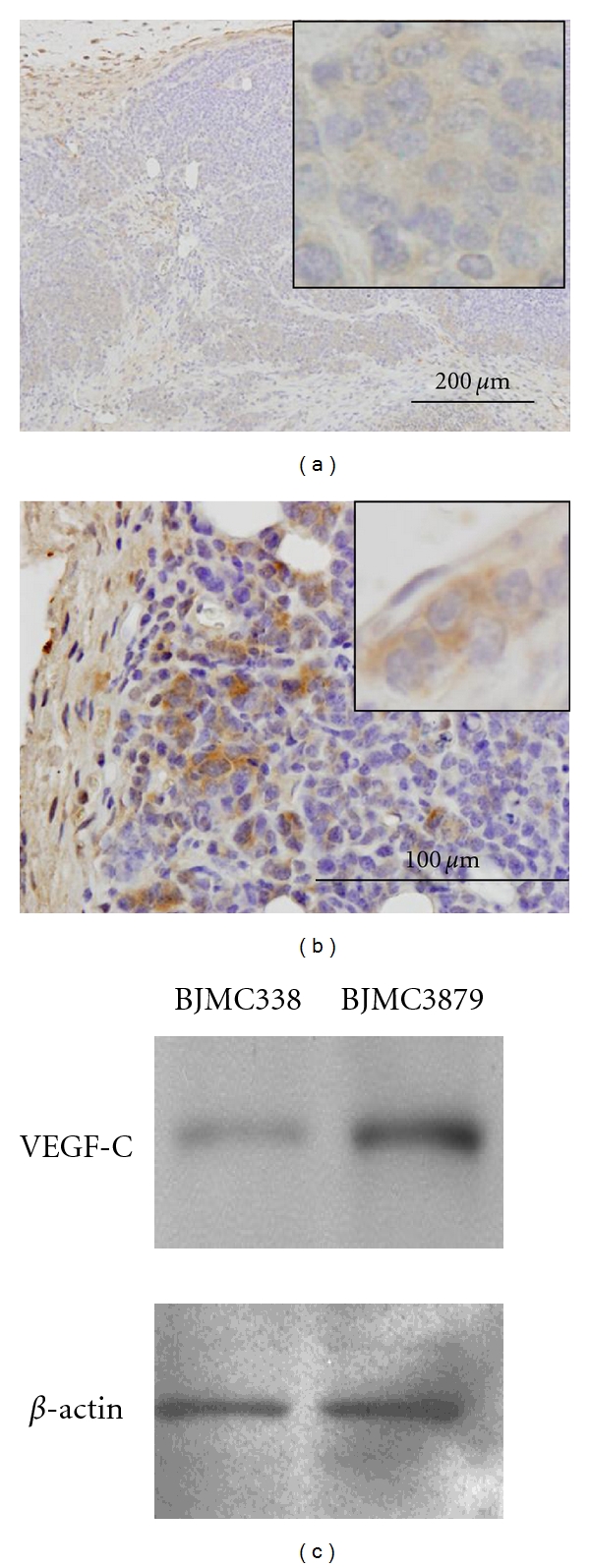
Immunohistochemistry (a and b) and Western blot analysis (c) of VEGF-C in BJMC338 tumor (a and c) and BJMC3879 tumor (b and c) at 8 weeks postinoculation.

 VEGF-C-positive cells were localized mainly in the peripheral viable regions of tumors. Immunohistochemical studies and Western blot analyses clearly demonstrated weak expression of VEGF-C in BJMC338 tumors (Figures 7(a) and 7(c)), relative to the strong expression in BJMC3879 tumors (Figures 7(b) and 7(c)).

#### 3.2.6. Distribution of CD68-Positive Macrophages in the Tumors

**Figure 8:**
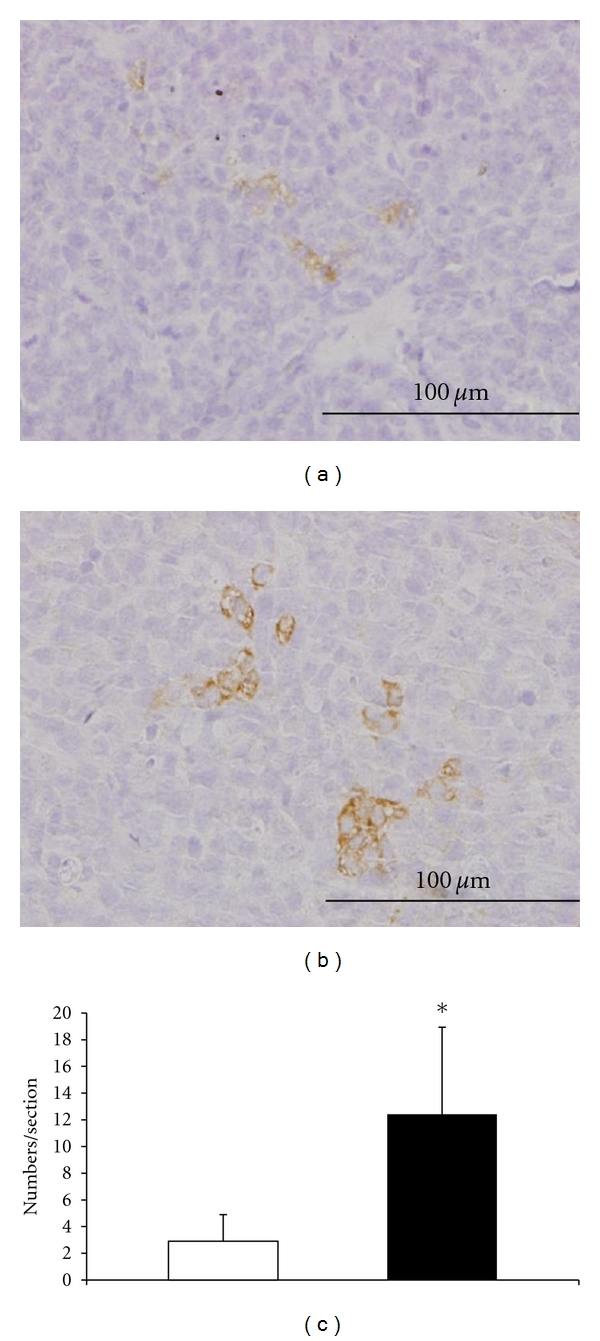
Immunohistochemistry of CD68 in BJMC338 tumor (a) and BJMC3879 tumor (b) at 4 weeks postinoculation. The density of macrophages in BJMC3879 tumors was significantly higher than that in BJMC338 tumors (*P < 0.001) (c).

 CD68-positive macrophages were found mainly in the viable regions of both tumors (Figure 8). CD68-positve macrophages containing vacuoles in their cytoplasm aggregated in the tumors. The density of macrophages in BJMC3879 tumors was significantly higher than that in BJMC338 tumors (*P* < 0.001) (Figure 8). 

## 4. Discussion

 In this study, we found that mouse mammary tumor cells (BJMC3879) that have high metastatic propensity expressed a higher level of VEGF-C than the mouse mammary tumor cells (BJMC338) with low metastatic propensity, and the inoculated BJMC3879 tumors expressed VEGF-C equivalently to tumor cell lines. In highly metastatic mouse mammary tumors (BJMC3879), LVD and the VEGF-C expression level were higher than those in the poorly metastatic mouse mammary tumors (BJMC338). BJMC3879 tumor cell inoculation resulted in axillary lymph node and lung metastases, whereas no metastasis occurred after BJMC338 tumor cell inoculation.

There are some clinical surveys of human breast cancer to prove a causal relationship between LVD and malignancy, the VEGF-C expression, lymph node metastasis, and prognosis [1, 4, 5]. Increased LVD in breast cancer was correlated with lymph node metastasis and VEGF-C expression. It was concluded that a high LVD may be a significant unfavorable prognostic factor for long-term survival of breast cancer patient. Our results correlate with their reports. Contrary to the clinical importance of these LVDs and on the basis of clinicopathological studies including breast cancers, it is the hypothesized that intratumoral lymphatics have no function. None of the breast carcinoma was found to contain Ki-67-positive dividing endothelial cells of lymph vessels [23], and by experimental microlymphangiography assay, no functional draining intratumoural lymphatics were found [24]. They also found that the functional lymphatics in the tumor margin alone were sufficient for lymphatic metastasis [24, 25]. It was reported that the degree of axillary lymph node metastasis increased in parallel with increasing LVD, patients with a high peritumoral LVD had only 58% 5-year distant disease-free survival as compared with 74% among those with a low peritumoral LVD. In addition, the presence of intratumoral lymph vessels was associated with neither axillary nodal status nor survival [24]. Moreover, not only LVD but also the size of peritumoral lymph vessels may be a significant consideration of lymph node metastasis [7]. As the enlarged lymphatics may collect interstitial fluid and cancer cells oozing from the tumor surface, it was suggested using mouse hybridoma cells and their syngenic mice that both peritumoral and intratumoral lymph vessels may play a crucial role in metastasis [26].

 VEGF-C expression in breast cancer has been considered as a clinicopathological prognostic factor [8, 27–29]. However, a univariate study by Bando et al. revealed that high VEGF-C expression level was significantly associated with a favorable prognosis for disease-free survival and overall survival (e.g., high VEGF-C levels were associated with low-grade tumors and a smaller size). Furthermore, multivariate analysis confirmed the independent prognostic value of VEGF-C [30]. Watanabe et al. compared the expressions of CD44 variants and VEGF-C as associated factors with long-term prognosis, they concluded that there was no association between VEGF-C expression and clinicopathological prognostic factor [31]. A clinicopathological study using RT-PCR indicated that VEGF-C and VEGF-D were involved in lymphatic vessel invasion prior to lymph node metastasis, and their expression level decreased after the occurrence of lymph node metastasis [32]. In a retrospective study of 61 cases, it was reported that LVD may serve as a predictor of lymph node metastasis and a prognostic factor, whereas VEGF-C and VEGF-D may play important roles in lymphangiogenesis, making the carcinoma more aggressive and leading to a poor prognosis in breast cancer [8]. Because of controversial results showing that high VEGF-C levels are associated with low-grade tumors and a smaller size, Bando et al. suggested that the mechanism of VEGF-C protein processing in human cancer requires further study [30]. Because intratumoral VEGF-C protein level changes in response to intratumoral microenvironments, the expressions of VEGF-C and VEGF-D may be inadequate as clinicopathological prognostic factors by themselves.

 To evaluate the effect of VEGF-C on lymphangiogenesis and lymph node metastasis, some human breast cancer cell lines were used *in vivo* and xenotransplanted to immunodeficient mice, SCID mice, or nude mice [11, 12]. Using human MCF-7 breast cancer cells, which are poorly invasive and estrogen dependent, Mattila et al. showed that tumor growth was stimulated *in vivo* in VEGF-C overexpressing MCF-7 cells xenotransplanted to nude mice. Furthermore, LVD in intra- and peritumoral lymphatic vessels was increased in tumors promoted by VEGF-C derived from xenotransplanted MCF-7 cells. While these reports, even though in xenotransplanted mice, support our results that tumoral VEGF-C expression plays an important role in lymphangiogenesis and lymph node metastasis of mouse mammary carcinoma, the role of immune cells including macrophages must be considered in tumor metastasis. Recent studies showed that VEGF-C is secreted by TAMs [13–15]. Our immunocompetent mouse models clearly showed the presence of CD68-positive TAMs in the inoculated tumors, and the density of TAMs in the high-metastatic mouse model was higher than that in the low-metastatic mouse model.

## 5. Conclusion

 The LVD in mammary carcinoma strongly expressing VEGF-C was higher than that in carcinoma expressing a low VEGF-C level with the former showing axillary lymph node metastasis. Our inoculated mouse mammary carcinoma models in this study are allotransplanted and immunocompetent tumors which show the same lymph node metastatic spectrum as human breast cancers. Consequently, our mouse models are the most ideal for the study of lymph node metastasis.

## Figures and Tables

**Table 1:** Number of mice which have lung and axially lymphnode metastases. Metastases were confirmed by H&E staining.

Lung	4W	6W	8W	10W
BJMC338	0	0	0	0
BJMC3879	0	0	5	5

Axially lymph node	4W	6W	8W	10W
BJMC338	0	0	0	0
BJMC3879	0	5	5	5
